# Altered negative priming in older subjects: first evidence from behavioral and neural level

**DOI:** 10.3389/fnhum.2012.00270

**Published:** 2012-10-01

**Authors:** Eva Bauer, Helge Gebhardt, Harald Gruppe, Bernd Gallhofer, Gebhard Sammer

**Affiliations:** Cognitive Neuroscience at the Centre for Psychiatry, Justus Liebig UniversityGiessen, Germany

**Keywords:** negative priming, aging, inhibition, fMRI, VBM

## Abstract

The impact of aging on the negative priming (NP) effect has been subject of many studies using behavioral measures. Results are inconsistent and corresponding neural data do not exist. We were interested in, whether or not processing of NP is altered in older in comparison to young adults (YA) on behavioral and neural level. Eighteen young and eighteen older healthy adults performed a location-based NP paradigm during fMRI. YA behaviorally showed a NP effect and NP associated fronto-striatal activation, which is in accordance with the inhibitory model of NP. In older subjects no significant behavioral NP effect and no NP-related activation in predefined brain regions could be found. This is discussed in context of the “loss of efficiency” hypothesis. One possible source for the lack of NP-related activation is a reduction of gray matter (GM) volume in older subjects as shown using voxel based morphometry (VBM).

## Introduction

Inhibition of irrelevant information has been found to be age sensitive (Hasher and Zacks, 1988; Dempster, 1992). This deficit may lead to interference of working memory contents by irrelevant information (Hasher et al., 1999) and therefore to increased distractibility from irrelevant stimuli (Connelly et al., 1991; Li et al., 1998; Alain and Woods, 1999; Sweeney et al., 2001; Juncos-Rabadan et al., 2008). Several tasks requiring inhibition of irrelevant stimuli (for example the Stroop task) are age sensitive (Van der Linden et al., 1994; Spieler et al., 1996; Klein et al., 1997; Wecker et al., 2000; Milham et al., 2002). However, Kramer et al. (1994) showed that the performance in some of the inhibitory tasks was impaired in older subjects, but not in all. In addition, other factors like the number of distracting stimuli or the distinguishability of targets from distractors influence the performance of older subjects in inhibitory tasks (Greenwood et al., 1993; Carlson et al., 1995; Scialfa et al., 1998; Hommel et al., 2004). Thus, inhibitory tasks are not fully comparable. Some appear to be age sensitive, others not. We are interested in, whether older adults (OA) show reduced ability in a location-based NP task, requiring inhibitory processes. This task is of particular interest, because NP is a task measuring inhibition in an indirect way, allowing drawing conclusions regarding neural processing of inhibition.

NP refers to increased response times observed in responding to a target, which has been presented as distractor immediately before (Tipper and Driver, 1988). Therefore, the NP effect indicates not only processing of attended information, but processing of ignored, inhibited information as well (Tipper, 1985). In a location-based NP task, the initial display of a trial (prime) consists of both, a distractor and a target stimulus. In the next display (probe), the target is presented at the location where the prime distractor was shown before. Inhibitory mechanisms are assumed to play a crucial role in the formation of the location-based NP effect (the selective inhibition model, Houghton and Tipper, 1994). Other mechanisms being involved in the processing of NP, like for example retrieval mechanisms, are also discussed (e.g., Neill et al., 1992).

Houghton and Tipper (1994) stated that target selection in the prime is based on persistent inhibition of the prime distractor^1^. With the end of the presentation of the prime, inhibition decays gradually, and is still active when the probe is subsequently presented. Those continuously activated inhibitory processes must be overcome, which is time consuming, and results in prolonged response times. A negatively primed probe target is a particularly “weak” target in comparison to a probe target of a control trial, because it is the same stimulus as the prime distractor, which was formerly inhibited (compare Frings and Groh-Bordin, 2007; Bauer et al., 2012). Thus, in NP trials increased inhibitory processes again suppress the predominant probe distractor. In addition to inhibition, response conflict can arise because the subject must treat an inhibition-assigned stimulus as target (Krueger et al., 2007). Several studies support the assumption that inhibitory processes are involved in the formation of the location-based NP effect on behavioral level (Tipper et al., 1990; Neill et al., 1994; Christie and Klein, 2001; Tipper, 2001; Buckolz et al., 2002; Vink et al., 2005; Guy and Buckolz, 2007). In an fMRI study, Bauer et al. (2012) found fronto-striatal activation in association with location-based NP. Studying 28 healthy subjects performing a location-based NP task, activation in the caudate nucleus (NC) and the anterior cingulate cortex (ACC) have been found. This supports the assumption that inhibitory processes are involved in the processing of the probe. Results of other imaging studies examining location-based NP also found neural evidence supporting the selective inhibition account (Wright et al., 2006; Krueger et al., 2007). Although the findings of these studies show very little convergence, all these studies consider their findings in accordance with the selective inhibition account. Wright et al. (2006) and Krueger et al. (2007) focused on attentional processes, Bauer et al. (2012) on inhibitory processes. In the present study, emphasis was put on inferior frontal areas, ACC and parts of the basal ganglia as target regions for location-based NP. These regions are known to be involved in inhibitory processes (Casey et al., 2001), in which we are especially interested. Moreover, they are consistent with our predictions regarding the inhibitory mechanisms involved in the processing of NP (compare Bauer et al., 2012).

There is evidence that retrieval mechanisms are also involved in processing of NP. Recently, theories combining both mechanisms have been suggested (May et al., 1995; Tipper, 2001; Neill, 2007). It may be best to assume that more than one mechanism is involved in the processing of NP. Factors have been discussed that influence the predominance of one of those mechanisms (for example May et al., 1995; Tipper, 2001; Neill, 2007). The presence of different processing mechanisms would be indicated by different brain activation patterns, e.g., retrieval mechanisms in hippocampal activation and inhibitory mechanisms in fronto-striatal activation. In this study we focus on fronto-striatal and ACC activation, which is commonly associated with inhibition and conflict and has been found in the context of location-based NP (Bauer et al., 2012).

To sum up, the presence of the NP effect indicates that—at least among others—inhibitory processes became active. NP measures inhibition indirectly, because subjects are not aware of the relevant response characteristics (Tipper, 2001). Although the task does not explicitly or consciously require inhibition like for example a GoNogo task, in young and healthy subjects the NP situation automatically triggers inhibitory mechanisms and therefore these subjects show a NP effect. Hence, the NP effect observed in location-based paradigms reflects inhibitory processes that normally take place in solving tasks.

Although there has been for years an ongoing discussion about the effects of aging on NP in general, location-based NP in association with aging has not been studied that extensively. An early review by Verhaeghen and De Meersman (1998) reported no effect of age for location-based NP, this means comparable NP effects for young and OA. Some recent studies support the findings of Verhaeghen and De Meersman (1998) and find a reliable location-based NP effect in OA (McAuliffe et al., 2006; Troche et al., 2008). However, decreased response times for location-based NP in older subjects have also been reported (Witthöft et al., 2009). This result is in agreement with the inhibitory deficit hypothesis (Hasher and Zacks, 1988; Hasher et al., 2007), claiming that inhibitory performance decreases with aging. According to this hypothesis, the main consequence of those OA' inefficient inhibitory processes is that irrelevant information gains entry into working memory, causing interference. In conclusion, the available data concerning the impact of age on location-based NP are not fully understood at present. Location-based NP might be one of the age sensitive tasks involving inhibition.

Since inferior frontal areas detoriate with advancing age (Raz, 2000) and have been related to inhibitory deficits in older subjects (West, 1996, 2000; Bugaiska et al., 2007), a decline of the inhibitory functions for older subjects can be explained (McDowd and Oseas-Kreger, 1991). Inferior frontal areas are associated with location-based NP, indicating altered location-based NP in older subjects.

In general, findings of recent studies investigating cognitive changes in OA on neural level can be classified in two rough categories. The “loss of efficiency hypothesis” summarizes patterns of reduced neural activation in regions engaged by young subjects coming along with no increased activation in other areas. On the other hand, “loss of selectivity” is discussed. This means less selectivity in recruitment of brain areas in form of additional activation in brain regions not activated by young adults (YA) (Reuter-Lorenz, 2002) to be characteristic for aging.

For inhibitory tasks like GoNogo and Flanker-tasks altered activation occurs with advancing age. Older compared to young subjects mostly show more extensive activations in frontal regions, as well as bilateral activation for correctly answered Nogo/incongruent Flanker trials (Nielson et al., 2002; Langenecker and Nielson, 2003; Colcombe et al., 2005). Behaviorally, YA and OA showed comparable performance for Nogo trials (not for Go trials) in the GoNogo tasks. For the Flanker task, OA showed proportionally more extensively prolonged response times for incongruent compared to congruent trials than YA. This is compatible with the “loss of selectivity hypothesis.” Alternatively, this activation pattern could reflect *compensatory* recruitment of brain areas to cope with neural dysfunction with advancing age. In the applied tasks compensation through effort on behavioral level may have influenced activation patterns. This means that increased neural activation in OA could be caused by making effort to reach good behavioral performance. NP tasks differ from those tasks in several key characteristics. Location-based NP is an *indirect* method to measure the magnitude of suppression of distractors, because subjects are not aware of the relevant response characteristics (Tipper, 2001). No compensation through effort on behavioral level is possible at all. Consequently, compensatory cognitive operations driven by an “effort bias” are not to be expected. Thus, additional brain activation in inhibition-associated regions in older subjects for location-based NP could serve as evidence for the loss of selectivity hypothesis. When additional brain activation occurs in other regions, not associated with inhibitory processes, like for example the hippocampus, the situation is different. Such activation patterns can have other causes than loss of neural selectivity. For example other processing mechanisms triggered by the NP situation like memory processes could be associated with those activation patterns.

Aging comes along with changes in gray matter (GM) volume. In a longitudinal study by Raz et al. (2005), shrinkage of brain volume was detected particularly in the NC, the cerebellum, the hippocampus and the association cortices. Only for the NC, sex differences in shrinkage were found. Neither brain size nor educational attainment had an influence on the volume changes (Raz et al., 2005). Several studies show decreased GM volume in the inferior frontal areas using voxel-based morphometry (VBM), (Ohnishi et al., 2001; Van Laere and Dierckx, 2001; Tisserand et al., 2002). Degeneration was also reported for striatal areas, especially the NC. A longitudinal comparison strengthened this finding; the average shrinkage rate of 0.83% per year for the NC is higher than those of other striatal areas (Raz et al., 2003).

It can be summarized that areas which are possibly associated with NP are deteriorating with advancing age. Hence, GM reduction with advancing age may be associated with the NP effect.

To our knowledge there are no papers investigating age-related changes in neural processing of location-based NP so far. This study aims to contribute to this issue based on the following assumptions. With recent findings on behavioral level, it is supposed that young subjects—contrary to older subjects—reliably show a location-based NP effect. For young subjects, fronto-striatal activations are to be expected on neural level for location-based NP. Due to the reports of an inhibitory deficit on behavioral level for OA, it can be assumed that reduced activation in brain regions associated with NP accompanies advancing age. This is in accordance with the loss of efficiency hypothesis. Further, we are interested in whether or not OA show additional activation in contralateral brain regions. A possible substrate of such a loss of efficiency could be due to the reduction of GM in brain regions, which are necessary for NP.

## Results

### Behavioral data

**Figure 1:**
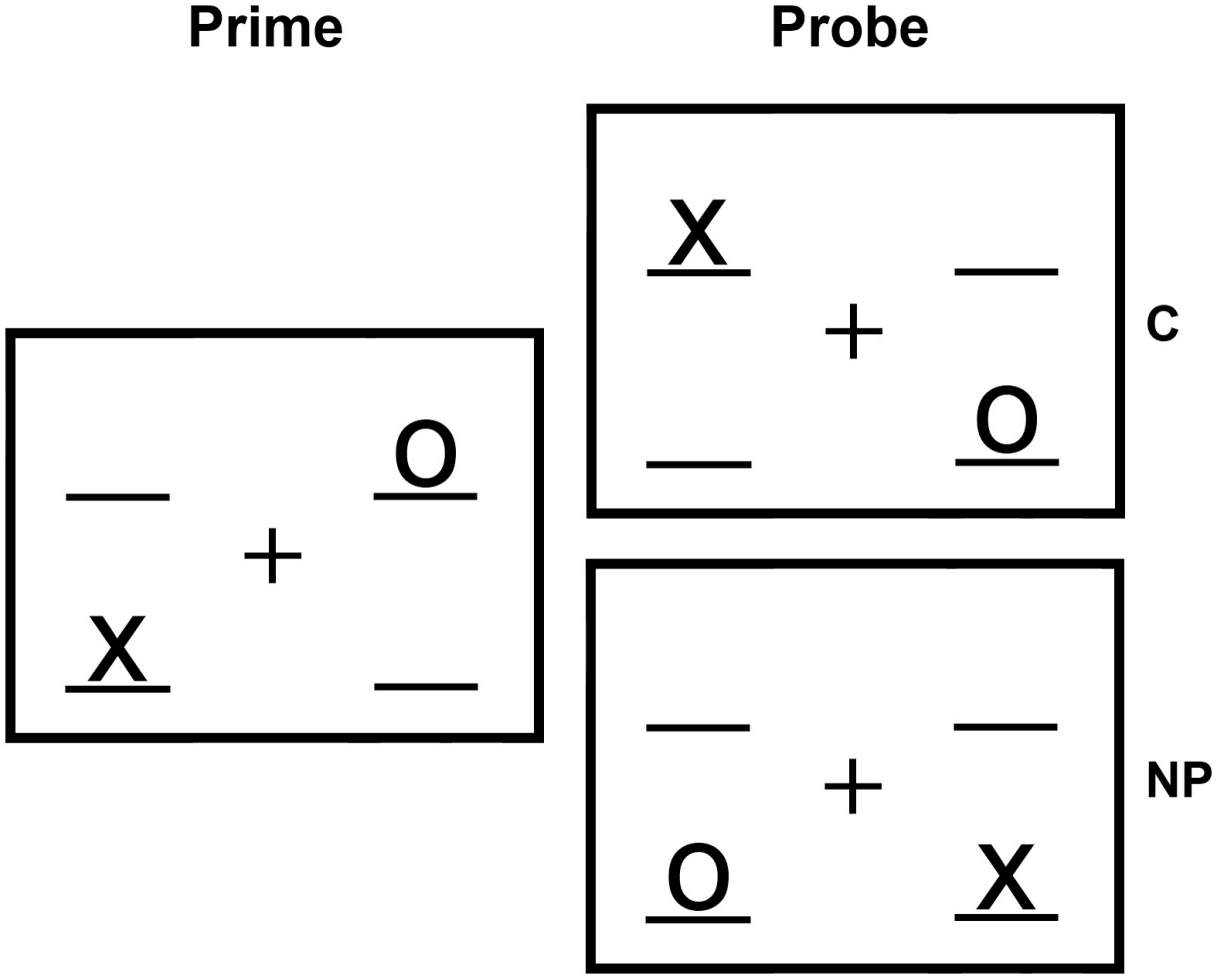
**Exemplary illustration of the experimental conditions used in the experiment.** In reality the numbers have been represented in light gray on a dark grey screen. In this example the circle is defined as target and the cross as distractor.

Examining two age groups, YA and OA, with a location-based NP paradigm including a negative priming (NP) and a control condition (C) in the scanner (Figure 1), we found significant differences in the basic response time between YA and OA [YA: 557 ms; OA: 704 ms; *t*_(34)_ = −3.50; *p* = 0.001]. Correlation between the basic response time and the NP effect was *r* = −0.50 (*p* = 0.002). The Levene Test revealed significant differences for variances of response times for C [YA: *V* = 34 ms; OA: *V* = 90 ms; *F*_(1, 34)_ = 5.99; *p* = 0.020] and for NP [YA: *V* = 29 ms; OA: *V* = 90 ms; *F*_(1, 34)_ = 5.85; *p* = 0.021] between the two age groups. For this reason we tested for a NP effect separately in each group using *t*-Tests. YA showed a significant NP effect [*t*_(17)_ = −6.09; *p* = 0.000]. For OA, no significant NP effect was found [*t*_(17)_ = −1.58; *p* = 0.13]; (Table 1).

**Table 1:** **Averaged response times in ms with the according standard-deviation (SD) for the conditions C and NP separately for young and older adults**.

	**Response time (SD)**
**C_YA_**	521.60 (93.30)
**NP_YA_**	542.48 (93.19)
**C_OA_**	683.32 (184.94)
**NP_OA_**	693.38 (171.20)

Note: YA, young adults; OA, older adults.

**Figure 2:**
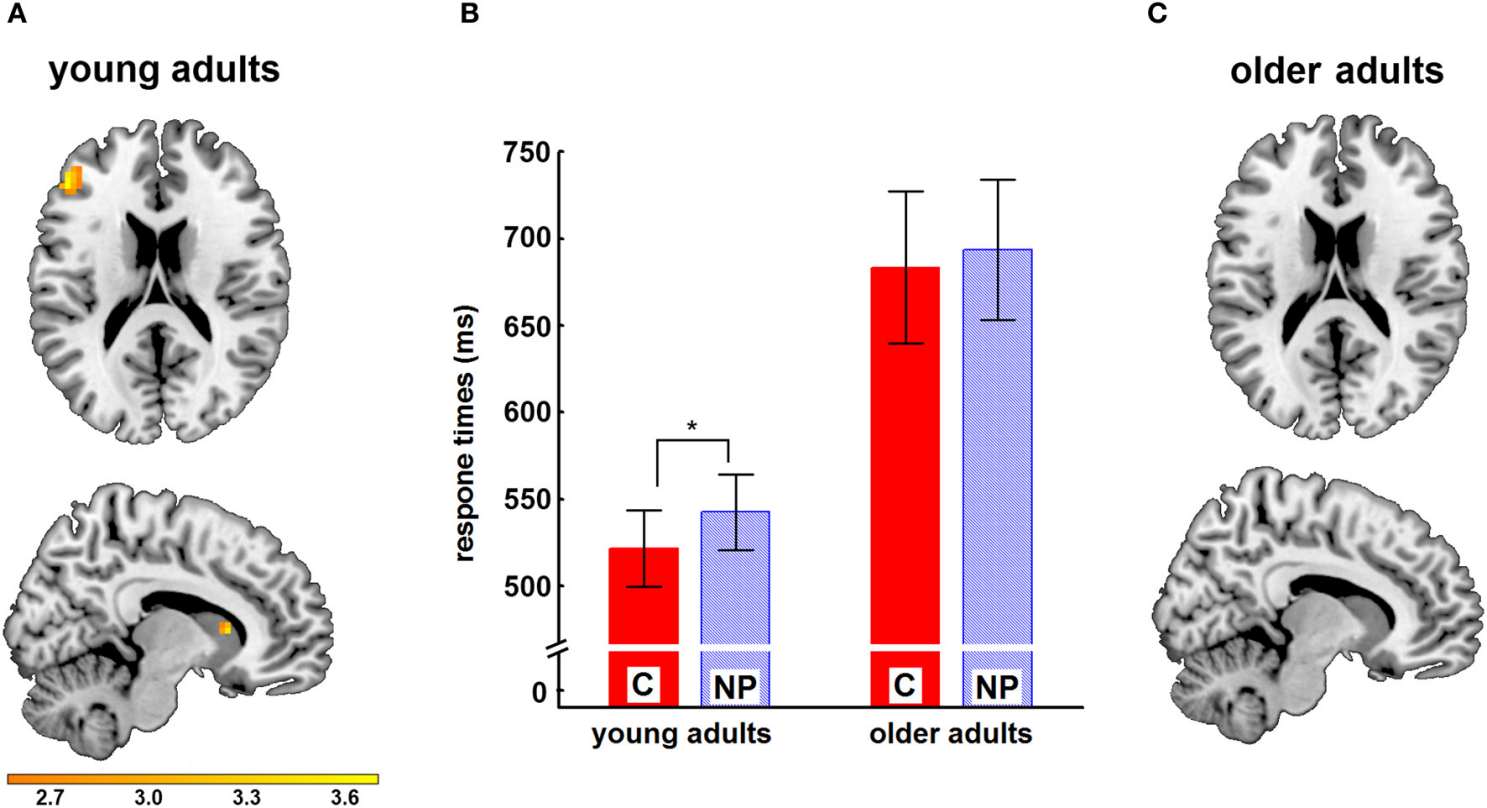
**Behavioral and neural results for young and older subjects.** In panel **(B)**, mean response times in ms and their standard errors for the conditions C (red bars) and NP (blue striped bars) are shown separately for young and older adults. ^*^marks significant differences (*p* = 0.000) between groups. Panel **(A)** shows neural activation for the contrast (NP–C) in young adults in the brain slice with *x* = 10 and *z* = 19, respectively. Activations in the frontal inferior gyrus and the caudate nucleus can be seen. Panel **(C)** shows the respective neural data for the older subjects. For illustration reasons only, data were thresholded at *t* ≥ 2.56, *p* = 0.05.

Figure 2 illustrates the differences between response times for C and NP in the two age groups, which are on average 20.88 ms for the young, and 10.06 ms for the older adults.

The averaged number of errors was very low in young [C: *M* = 0.33 (SD = 0.84), NP: *M* = 0.28 (SD = 0.75)] and older adults [C: *M* = 1.22 (SD = 1.73), NP: *M* = 0.72 (SD = 1.07)]. The total number of errors did not differ between groups (YA: *M* = 0.61; OA: *M* = 1.94; *z*_*corr*_ = 1.18; *p* = 0.24).

### Functional imaging data

Neural data were collected using fMRI. Subtracting C from NP revealed significant activations in frontal and striatal regions in the group of YA (Table 2, Figure 2). In the group of OA, for this contrast no significant activations in any selected region of interest (ROI, see section “fMRI Data Analysis”) could be found. Adjacently, we computed the contrasts [(NP_YA_–C_YA_)–(NP_OA_–C_OA_)] and [(NP_OA_–C_OA_)–(NP_YA_–C_YA_)] to investigate, whether NP is processed differently in older compared to YA. Hemodynamic activation was found in the NC for the contrast [(NP_YA_–C_YA_)–(NP_OA_–C_OA_)]. Again, the reverse contrast did not result in any significant activation. The basic response time was expectedly significantly higher in OA than in YA [compare section “Behavioral Data”]. Therefore the same analysis was undertaken introducing the basic response time as a covariate to ensure that possible neural age effects were not driven by differences in basic response time between both groups. For those analyses similar results with small changes in *t*- and *p*-values were obtained (see Table 2).

**Table 2:** **Localization and statistics of the peak voxels within the respective ROI for the contrast (NP – C) in young adults and older adults separately, and for the contrast (NP – C) in older adults subtracted from the contrast (NP – C) in young adults and vice versa**.

**Contrast**	**Brain structure**	***x***	***y***	***z***	***t***	***p*_corr_**
**NP_YA_–C_YA_**	R caudate nucleus head/body	9	17	7	3.47	0.018
	L BA 46 (inferior frontal p.t.)	−48	35	19	3.70	0.035
	*R caudate nucleus head/body*	*9*	*17*	*7*	*3.49*	*0.020*
	*L BA 46 (inferior frontal p.t.)*	*−48*	*38*	*19*	*3.69*	*0.041*
**NP_OA_–C_OA_**	n.s.					
**[(NP_YA_–C_YA_)–**	R caudate nucleus head	12	17	−5	3.14	0.020
**(NP_OA_–C_OA_)]**	*R caudate nucleus head*	*12*	*17*	−*5*	*3.21*	*0.018*
**[(NP_OA_–C_OA_)–(NP_YA_–C_YA_)]**	n.s.	‒	‒	‒	‒	‒

Results of the additional analysis including basic response time as covariate are written in italics.

Note: The threshold was p_corr_ < 0.05 (FWE-corrected according to SPM8, small volume corrected). All coordinates (x, y, z) are given in MNI space. L, left; R, right; n.s., no significances revealed; YA, young adults; OA, older adults; p.t., pars triangularis.

In accordance with behavioral data, we found differences in processing of NP for YA and OA on neural level.

### Structural imaging data

**Figure 3:**
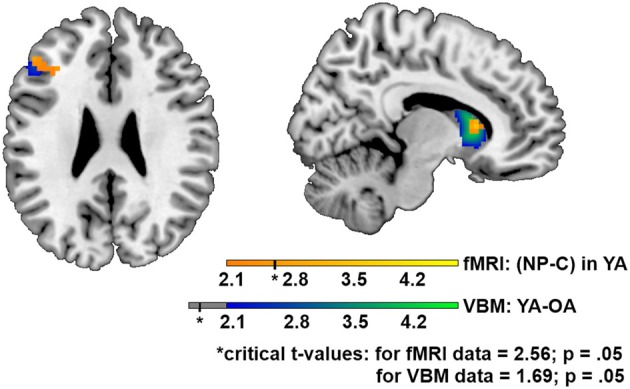
**Differences in gray matter volume between young and older subjects as well as NP associated brain activation in young subjects.** GM volume for the contrast (young adults–older adults) is presented in blue/green color, NP associated brain activation in young subjects (NP_YA_–C_YA_) in orange/yellow. Brain slice with *z* = 25 (left) and *x* = 10 (right) are shown. Differences in the frontal inferior gyrus and the caudate nucleus can be seen.

Addressing structural differences in functional relevant regions, we applied VBM. Herewith, we examined GM volume differences with a whole brain (WB) analysis, and in NP relevant brain regions using an ROI approach. In the WB analysis, OA in comparison to YA showed reduced GM volume in the right inferior frontal pars triangularis (bordering the insula) and in the cerebellum. For the ROI analysis we chose the left BA46 and the right NC (head) according to the fMRI results of the contrast (NP_YA_–C_YA_). In the ROI analysis, we found significant GM changes in the NC, and tendencies in the BA46 for the contrast (YA–OA), (see Table 3, Figure 3). A positive correlation between GM volume and behavioral NP effect was found for the right NC (head).

**Table 3:** **Localization and statistics of the peak voxels for the GM volume analysis for (YA – OA) and for the positive correlation of GM volume with the NP effect**.

**Analysis**	**Brain structure**	***x***	***y***	***z***	***t***	***p*_corr_**
(YA–OA)	*R inferior frontal p.t./ insula*	*45*	*20*	−*5*	*6.86*	*0.005*
	*R cerebellum*	*30*	−*66*	−*30*	*6.58*	*0.009*
	*L cerebellum*	−*23*	−*75*	−*23*	*6.36*	*0.016*
	L BA46 (inferior frontal p.t.)	−50	11	34	4.10	0.073
	R caudate nucleus head	10	17	6	4.19	0.003
Correlation	R caudate nucleus head	9	17	−5	3.10	0.038

Results of the WB analysis are written in italics.

Note: The threshold was p_corr_ < 0.05 (FWE-corrected according to SPM8, small volume corrected). All coordinates (x, y, z) are given in MNI space. L, left; R, right; p.t., pars triangularis.

Consistent with our hypothesis, we found GM volume reduction for older adults in regions, which are associated with NP in YA.

## Discussion

### Behavioral data

A well-established location-based NP paradigm was applied in YA and OA during fMRI. On behavioral level, a significant NP effect was found in the young, but not in the older subjects. This confirms recent behavioral studies investigating location-based NP (Witthöft et al., 2009). Because of the characteristics of location-based NP as described in the introduction, NP reflects inhibitory processes as normally used in solving tasks. That means although the task does not explicitly/consciously require (cognitive) inhibition, in young healthy subjects the NP situation automatically triggers inhibitory mechanisms and results in a significant NP effect. Thus, the task reflects the automatic use of inhibition for task solving. In this regard, tasks which elicit location-based NP are very sensitive. This may explain the inconsistency of the findings for location-based NP in OA in literature to some extent.

### Functional imaging

On neural level, the young group showed activation in fronto-striatal areas. Activation in those areas was frequently related to processes of inhibition applying diverse tasks (Rubia et al., 2007). Fronto-striatal activation also has been described for processing location-based NP (Bauer et al., 2012). Thus, the data of the present study are in accordance with recent findings and theories of location-based NP. Inhibition of the prime distractor and the resulting strong probe distractor result in prolonged response times for NP situations and trigger fronto-striatal activation.

Testing the contrast (NP–C) in the older group, we did not find significant NP-related activation in the a priori defined ROI. Additional support comes from the finding that only young subjects show greater NP-related activation compared to older subjects in predefined areas, but not vice versa. Analyses including basic response times as covariate indicated that differences between age groups are not driven by differences in basic response times. Our results are clearly not in line with the bilateral activation and overactivation patterns, which have been found in several other inhibitory tasks for older subjects (Nielson et al., 2002; Langenecker and Nielson, 2003; Colcombe et al., 2005). Contrary to those tasks, compensation through effort on behavioral level does not have any effect on the occurrence of the NP effect. Gaining good behavioral performance by compensatory effort, which might have influenced activation patterns in other inhibitory tasks reported elsewhere, can clearly be excluded as confounding variable in this experiment. The missing fronto-striatal inhibition-associated activation in OA indicates a loss of neural efficiency and not loss of neural selectivity or compensatory recruitment of additional brain activation for the processing of NP. Such a finding is only reliably detectable in tasks where compensation through effort is made impossible. However, the ROI analyses were designed to reveal activation differences in inhibition-associated areas, but not to find activation differences in regions associated with alternative processes, like e.g., memory processes or others. Therefore, differences in other regions were solely tested using whole-brain analyses. We can not preclude age differences regarding other mechanisms than inhibition.

Logan et al. (2002) proposed that underactivation of brain areas probably develops in the 6th decade of life, whereas overactivation (in a compensatory or non-selective sense) may develop in the 7th or 8th decade of life. Since only two out of 18 OA were over 70 years old (mean age: 60.17; SD = 6.20; Range: 54–77) in this study, the data data fit very well with the observations of Logan et al. (2002). All in all, our results can be embedded best in the theoretical framework of the inhibitory deficit model. This model states that inefficient inhibitory processes of OA result in interference, because irrelevant information gains entry into working memory (Hasher and Zacks, 1988; Hasher et al., 2007). In location-based NP tasks, the inhibition of the prime distractor impedes the processing of the subsequent stimuli (probe stimuli), and facilitates the processing of the actual target (prime target). Hence, the lack of the behavioral NP effect we observed in OA indicates that the probe distractor has not been inhibited effectively.

In this study we did not aim to study the role of decay of inhibition. However, decay of inhibition also might contribute or even lead to the altered processing of NP situations in OA. Experimental variation of the time window between the response to the prime and the presentation of the probe in YA and OA would allow the investigation of this question. Another limitation of this study results from the unequal distribution of the behavioral NP effect over both age groups. It reduces the possibility to differentiate between brain activation associated with age or performance in the NP task. Additional analyses to clarify this issue favor age- rather than performance-related effects. However, due to small sample size with increased number of cells of the resulting design these additional analyses lack statistical power and consist of further statistical problems. Further studies particularly designed to differentiate between performance- and age-related brain activation would be of great value. To be able to draw more general conclusions, further work has to be put on the question, whether or not loss of neural efficiency also is responsible for altered performance for all the various types of inhibition. For a memory task, in which compensation through effort on behavioral level was impossible, loss of neural efficiency in OA has been reported, too (Johnson et al., 2004).

### Structural imaging

Potential causes for the observed loss of neural efficiency are also of interest. Several potential candidates are discussed in the following. On the one hand, reduced signal amplitude could be a reason for the smaller activation. On the other hand, enhanced “noise” due to higher variability in brain activation in the sample could play a role. Reduced signal amplitude could occur due to well known vascular or neural changes with advancing age. GM volume tended to be reduced in older subjects in the left inferior frontal region, lying subjacent to the NP relevant frontal area (left BA 46). This is in agreement with findings showing that frontal areas are seriously affected by aging processes (Tisserand et al., 2002). Especially important in the current study is the role of the NC-head. For this region, next to significant GM volume reduction in OA, a direct relationship to the performance in the NP task was found. The size of the behavioral NP effect correlated with the GM volume. Hence, the NC, which is one of the core regions of the striatum, which in turn is affected by aging processes (Raz et al., 2003), is likely to play an important role in the context of NP in OA. Contrary to other studies investigating inhibition, we did not find compensating contralateral activation for NP. In the VBM analysis we found strong GM reduction in the right inferior frontal cortex (triangular part) applying a WB approach. Thus, in addition to the characteristics of NP, compensating recruitment of the contralateral hemisphere might not have been possible in this case, because that brain area as well was affected by significant atrophy. We additionally examined whether neural “noise” is of importance in this context. A Levene test was calculated to reveal whether the variability in the contrast estimates for the YA and OA differed significantly in the left BA 46 (*x* = −48, *y* = 35, *z* = 9) and in the right NC (*x* = 9, *y* = 17, *z* = 7). Results indicate no significant differences in variance [for the left BA 46: *F*_(1, 34)_ = 0.33; *p* = 0.57; for the right NC: *F*_(1, 34)_ = 0.003; *p* = 0.95].

In summary, the observed GM volume reduction in NP relevant areas, especially in the NC, in older subjects might contribute to their neural inefficiency in this task.

## Materials and methods

### Ethics statement

The study was approved by the Institutional Review Board of the University of Giessen; procedures and measures were explained to the participants who provided informed consent before participating in the study. The declaration of Helsinki was conformed.

### Participants

The sample consisted of 18 young healthy adults (group YA; mean age: 24.39; SD = 2.23; Range: 19–28) and 18 older healthy subjects (group OA; mean age: 60.17; SD = 6.20; Range: 54–77). A Wald–Wolfowitz-Test revealed that the two groups did not differ with respect to sex (*p* = 0.09), education (*p* = 0.50), being member of an academic community (*p* = 0.31) or being member of an actively working community (*p* = 0.18). Thus, we can assume that group differences are not driven by educational issues or sex. None of the older subjects suffered from dementia or depression. This was tested with the German version of the CERADplus (Morris et al., 1989; available online at: URL: http://www.memoryclinic.ch/content/view/19/51/) and the Geriatric Depression Scale (Yesavage et al., 1982; available online at: URL: http://www.stanford.edu/~yesavage/German.html). The study subjects received an expense allowance of 8 € per hour.

The anatomical scan of one healthy subject (30 year old female) which was excluded from the fMRI part of the study due to missing data, was included to build templates for VBM.

### Experimental paradigm

There exist diverse NP designs, which differ in several factors including timing, stimulus characteristics, and feedback. Only for few of those parameters explicit recommendations can be found in the literature. In our design we accounted for research results as well as for the examined sample and for our measuring instrument (fMRI).

The NP experiment began with a practicing session including five trials outside the MR examination cabin. The investigator gave oral feedback about the correctness of the given responses. During the NP task, the display was divided into four compartments, one of them containing a target and another distractor stimulus—in each case either a circle or a cross. Participants were asked to indicate the position of the target by pressing the corresponding button on a 4-button keypad with a holdover key in the middle. They were instructed to respond fast, but accurate, and to only use the index finger of their dominant (right) hand to press the button. The buttons on the keypad were arranged according to the compartments on the display. The experiment consisted of 144 trials, of which 72 corresponded to the control condition and 72 to the NP condition. Each trial started with a prime. As soon as the subject had pressed one of the four buttons (limited to 6000 ms), a fixation cross followed for 400 ms (compare Troche et al., 2009). Subsequently, the corresponding probe was presented in the same manner as the prime (disappearance with button-press, maximum 6000 ms appearance). Finally a fixation-cross was displayed for 2000–4000 ms.

Trials, in which the probe target was shown at a location that has been empty during prime presentation, formed the control condition. In the NP condition, the location of the prime distractor became the location of the probe target. In 50% of the NP conditions the probe distractor was shown at the location of the prime target (see Figure 1)^2^. Subjects were assigned to two different pseudo-random trial sequences consisting of equally distributed conditions over the time course of the task (restriction: no more than three trials of the same condition were allowed). For nine young and nine older subjects the circle was defined as target stimulus, for nine YA and nine OA the cross.

The entire procedure amounted up to 15 min, depending on the subject's speed of operation. The task presentation was controlled by Presentation software package (Neurobehavioral Systems, Albany, CA). Stimuli were projected on a backlight screen mounted near the MRT tube opening. Subjects were able to watch the screen by use of a head coil mounted mirror located approximately 20 cm above their eyes.

### Data acquisition

Imaging data were acquired by a 1.5 T whole-body tomograph (General Electric; MR Signa NV/I). To measure the blood oxygen level dependent (BOLD) contrast, a T2^*^-weighted single shot gradient echo EPI sequence (TR = 3 s, TE = 50 ms, flip angle = 90°, *FOV* = 240 × 240 mm^2^, 64 × 64 matrix) was used. Each volume contained 30 slices with 5 mm slice thickness. The slices were acquired interleaved, in ascending order.

Structural image acquisition consisted of T1-weighted sagittal images [MPRage, 1.6 mm slice thickness, TR = 13 ms, TE = 4.2 ms, flip angle = 15°, field of view = 240 mm × 180 mm, acquisition matrix 256 × 256, 172 slices, NEX (number of acquisition) = 1].

### Behavioral data analysis

Behavioral data were analyzed using the statistical software package Statistica 9 (StatSoft(Europe) GmbH, Hamburg). Basic response time, defined as median response latencies (from stimulus onset until response) over all prime stimuli, was calculated for each subject. Median was used on subject level, because the usage of medians copes with the left-sided distribution of reaction times. On group level, the average of the median response latencies was built. To analyze whether the two age groups differed in basic response times a *t*-test was computed.

For the analysis of response times with regard to the NP effect, only correctly answered trials were considered. Median response times (from stimulus onset until response) were calculated for the probe stimuli for each subject. For the analysis of main and interaction effects, we planned a Two-Way ANOVA (two age groups: YA and OA; two conditions: NP and C) for repeated measures. However, a Levene test indicated that the assumptions to conduct an ANOVA were not fulfilled, because the variance in the two age-groups differed significantly. For this reason we computed separate *t*-tests in each age-group to test for significant NP effects. Correlation analysis was conducted for basic response time and the calculated NP effect to reveal whether the NP effect is influenced by the basic response time. The behavioral NP effect was calculated as follows: (response to negatively primed probe—onset negatively primed probe)—(response to control probe—onset control probe); (compare Frings and Wentura, 2008). Whether the total number of errors differed among the groups was tested with a Wald–Wolfowitz-Test. For all statistical analyses α-threshold was set to *p* = 0.05.

### fMRI data analysis

fMRI data were analyzed using statistical parametric mapping methods with the SPM8 software package (Wellcome Department of Cognitive Neurology, London, UK) implemented in Matlab (Mathworks Inc., Sherborn, MA, USA). The first four volumes were discarded due to an incomplete steady state of magnetization.

Preprocessing consisted of slice time correction (reference slice: 29), realignment (2nd degree b-spline interpolation to the mean image), and normalization to the standard space of the Montreal Neurological Institute (MNI) brain. Spatial smoothing was applied using an isotropic three-dimensional Gaussian filter with a full width at half maximum (FWHM) of 8 mm to allow for corrected statistical inference.

The evoked BOLD responses were modeled for both conditions, C and NP. For an appropriate modeling of experimental conditions the exact durations of each single event was used. The beginning was set to the presentation of the prime stimuli, the ending to the individual response to the probe stimuli. Only correctly answered trials were included in the analysis. We used a canonical hemodynamic response function (SPM) without time and dispersion derivations for response modeling. In order to account for movement related variance six movement parameters derived from the realignment preprocessing step were included as covariates in the analysis. A high pass filter (time constant = 128 s) was implemented by using cosine functions in the design matrix.

WB analyses revealed no significant results for *p*_corr_ < 0.05, *k* = 0, family-wise error (FWE) corrected. ROI analyses were conducted with brain regions, which are significantly associated with the processing of NP (Bauer et al., 2012). We used the following structural ROI of the TD brodmann areas + atlas: frontal areas (BA 24, BA 44–BA 46), as well as putamen, pallidum (medial and lateral part), and NC (subdivided in head and body). All these analyses were conducted separately for each hemisphere. ROI masks were generated using the WFU PickAtlas, an automated software toolbox for generating ROI masks based on the Talairach Daemon database (Lancaster et al., 1997, 2000; Talairach and Tournoux, 1988; Maldjian et al., 2003). The PickAtlas automatically implements the SPM small volume correction, decreasing the number of voxels going into a multiple comparisons computation based on the size of the mask. All reported ROI results were tested at *p*_corr_ < 0.05 and adjusted according to the gaussian random field theory to control for the FWE. For ROI analysis all *t*-values and FWE corrected *p*-values are listed.

According to our hypotheses that location-based NP is processed by a fronto-striatal network, a one-sample *t*-test including the young group (NP_YA_–C_YA_) was conducted for the according ROI. The same contrast was also computed for the older group (NP_OA_–C_OA_) to learn about the processing of NP in this group. To analyze if one of the two age groups shows stronger activation for NP than the other group, we computed a two-sample *t*-test for the contrast (NP–C), in short [(NP_YA_–C_YA_)–(NP_OA_–C_OA_)] and [(NP_OA_–C_OA_)–(NP_YA_–C_YA_)]. The basic response time was expectedly significantly higher in OA than in YA (compare section “Behavioral Data”). To ensure that possible neural age effects are not driven by this difference in YA and OA, all contrasts were additionally calculated with the basic response time as covariate. For one-sample *t*-tests, the individual average response time for probe control trials was included as covariate. For the two-sample *t*-test, we implemented those averaged response times as changing covariate. This model assumes that the covariate changes within groups with the dependent variable, the BOLD, across levels of the independent variable. The change in the covariate is correlated with the change in the dependent variable and afterwards, the residual variance is analyzed in a standard two-sample *t*-test.

### VBM data analysis

#### Data processing

To perform VBM, all MR structural image data were processed using Statistical Parametric Mapping 8 (SPM 8) (http://www.fil.ion.ucl.ac.uk/spm/) running under MATLAB 7.1 (The Mathworks, Natick, MA, USA) (Ashburner and Friston, 2000). Before preprocessing, all the structural images were inspected for artifacts and the origin of the images was set at the anterior commissure. For preprocessing, DARTEL was used to improve inter-subject registration of structural images. The following processing steps were carried out: (1) GM, white matter (WM) and cerebrospinal fluid images (CSF) were generated using the standard unified segmentation model in SPM 8 (Ashburner and Friston, 2005). (2) Following this, GM population templates were generated from the entire image dataset, consisting of 37 subjects. Only 36 of them were included in the further analysis. Therefore the diffeomorphic anatomical registration using exponentiated Lie algebra (DARTEL) technique was used (Ashburner, 2007). (3) In a third step, an initial affine registration of the GM DARTEL templates to the tissue probability maps in MNI space (http://www.mni.mcgill.ca/) was conducted. Non-linear warping of GM images was performed to the DARTEL GM template in MNI space subsequently. (4) Images were modulated to preserve relative volumes of GM following the spatial normalization procedure. (5) In a final step, images were smoothed with an 8 mm FWHM Gaussian kernel. Preprocessed data of 36 subjects were used for statistical analysis.

#### Statistical analysis

All GM volume analyses were assessed using the general linear model in SPM8. Gaussian random fields theory was applied to estimate significance of each effect (Friston et al., 1994). Total intracranial volume, measured with SPM8, was used as globals; values of the preprocessed data were proportionally to the fraction of brain volume accounted for by that piece of GM. WB as well as ROI analyses were performed to investigate differences of GM volume between the two age groups. ROI were chosen according to the results of the fMRI part in order to study NP relevant areas. Comparable to the fMRI data analysis, ROI masks of the TD Brodmann areas + atlas were used. SPM small volume correction was applied. Frontal masks were slightly smoothed with a kernel of 2 mm because GM reduction in the border area was of interest with regards to content in this case. In addition, correlation between the GM loss and the behavioral NP effect was studied. Results were assessed using the FWE threshold of *p*_FWE_ = 0.05. Tendencies are also reported.

## Conclusion

We found that OA do not show activation patterns comparable to YA for location-based NP, which is in accordance with the behavioral data. These results fit with the model of neural inefficiency with advancing age. Based on the results of this study a loss of signal associated with a reduction of GM volume is proposed, but other processes may certainly also contribute.

### Conflict of interest statement

The authors declare that the research was conducted in the absence of any commercial or financial relationships that could be construed as a potential conflict of interest.

## Footnotes

^1^The subject of inhibition, that means whether the response, the perceptual pattern or the representation is inhibited, is still discussed.

^2^Bauer et al. (2012) showed that there is no significant difference on neural level between NP trials in which the probe distractor is shown at the location of the prime targets compared to trials where the probe distractor is presented on previously unoccupied locations.
